# Shifting from Fragmentation to Integration: A Systematic Analysis of Long-Term Care Insurance Policies in China

**DOI:** 10.5334/ijic.5676

**Published:** 2021-09-17

**Authors:** Wusi Zhou, Weidong Dai

**Affiliations:** 1 School of Public Administration, Hangzhou Normal University, China; 2 School of Public Administration, Zhejiang University of Finance and Economics, China

**Keywords:** older people, disability, long-term care, policy fragmentation, policy integration

## Abstract

**Introduction:**

Long-term care is an effective intervention that help older people cope with significant declines in capacity. The growing demand for long-term care signals a new social risk and has been given a higher political priority in China. In 2016, 15 local authorities have been selected to pilot the long-term care insurance programme. However, the current implementation of these programmes is fragmented, with a measure of uncertainty. This study aims to investigate the principles and characteristics of long-term care insurance policies across all pilot authorities. It seeks to examine the design of local long-term care insurance systems and their current status.

**Methodology:**

Based on the 2016 guidance, a systematic search for local policy documents on long-term care insurance across the 15 authorities was undertaken, followed by critical analysis to extract policy value and distinctive features in the delivery of long-term care.

**Results:**

The results found that there were many inconsistencies in long-term care policies across local areas, leading to substantial variations in services to the beneficiaries, funding sources, benefit package, supply options and partnership working. Policy fragmentation has brought the postcode lottery and continued inequity for long-term care.

**Discussion:**

Moving forward, local authorities need to have a clear vision of inter-organisational collaboration from the macro to the micro levels in directional and functional dimensions. At the national level, vertical governance should be interacted to outline good practice guidelines and build right service infrastructure. At the local level, horizontal organizations can collaborate to achieve an effective and efficient delivery of long-term care.

## Introduction

Aging population and associated disabilities have posed significant challenges to the sustainability of the healthcare system in China. According to the official statistics [1], there were about 33 million older people with partial or permanent disability in 2010, accounting for 19% of the total older population; among this, around 11 million (6%) were permanently disabled older population. The figures went up to over 40 million in 2015 and is projected by World Health Organization (WHO) to grow more quickly to reach 66 million in 2050 [2]. Disability trend in the elderly has placed serious impact upon the long-term care (LTC) system. LTC refers to a variety of healthcare services designed to assist people with disabling conditions in performing basic daily activities [3]. From the biological perspective, the aging process represents an accumulation of damages to cells and tissues over time [4]. This leads to a steady decline in physical and mental capacities as well as an enhanced vulnerability to infectious disease and chronic illness [5]. In 2013, there were nearly 50% of older people struggling with chronic diseases, and 37% of them experienced rapid deterioration in functional abilities [6]. According to projections, by 2030 older populations with one or more chronic illness can triple the number and nearly 80% of 60 years old and over will die from chronic diseases [2]. This dramatic increase requires a large amount of LTC, which indicates that the traditional family-oriented care system is unlikely to deliver them. Indeed, the increased needs for LTC have become a social risk [7]. Besides, influenced by traditional nursing home management systems, nursing institutions nowadays mainly provide daily care for older people with a severe shortage of other services such as recovery support, health maintenance, mental health and hospice care [8].

In response to these concerns, Chinese government introduced major reforms in the traditional aged service system by summarising experiences of German, Japan and South Korea’s practices on long-term care insurance (LTCI) [9]. In June 2016, the Ministry of Human Resources and Social Security of P.R. China issued *guidance on establishing long-term care insurance system in the pilot cities* [10]. This guidance named 15 pilot cities to develop their LTCI system and to use social insurance as a source of financing LTC services. Commercial insurance was not adopted as it only benefits a few people— in other words, public governance and its policies are the key solution to the difficulty of facilitating high quality LTC [11,12]. Therefore, it is necessary to build a formal LTCI system after a period of implementing temporary care service polices [13]. As evidenced, the earlier LTCI is introduced, the better social effect is produced [14].

Despite the 2016 guidance, different pilot authorities adopted different policies and practices on LTCI. Therefore, there is a need to investigate the current status of the LTCI programmes across the country. This study is aimed at reviewing and assessing the performance and effectiveness of the LTCI policy regime in China. It will seek to address three research questions: (i) what policies each pilot authority has adopted to carry out LTCI; (ii) what are main features of and common issues with current LTCI policies; (iii) how to integrate these fragmented policies for improvement of the LTCI system at both national and local levels.

## Integrating policies for LTCI: main rationales and theoretical framework

The LTCI system is defined as an insurance institution that offers daily life care, healthcare services and psychological help to disabled older people [15]. It normally consists of four dimensions: service beneficiaries, financing decisions, benefit package and service providers [16]. However, due to a lack of the united model, different programmes that supported specific ways of delivering LTC in different regions were implemented, causing policy fragmentation with their own characteristics in essential dimensions [17] as well as diverse influences on disabled people and their caregivers [7].

Firstly, service coverage and programme beneficiaries of LTC are restricted to certain members in each country. The LTCI system is designed to meet the escalating needs of older populations with chronic diseases or other disabilities [22,23]. However, because of differences in care needs between persons and in economic prosperity between and within countries, various LTCI policies were introduced to cover particular persons who meets the eligibility criteria [24,25]. By contrast, only a small number of older people have been insured within each country. For example, LTCI services covered just 5.8% of older population in South Korea, compared with 11% in OECD countries, 14.5% in Germany and 18.5% in Japan [26]. Service beneficiary is another key feature of the LTCI policy, which decides whether these vulnerable groups are qualified for LTC services and better life quality [27]. Unfortunately, due to bureaucratic obstruction and resource shortage, there were always some older people with critical conditions beyond coverage of the LTCI programme in many countries such as South Korea [28,29].

Secondly, funding sources and payment rates are found variably across the world. In principle, LTC services should be financed by multiple sources, with public funds making a dominant contribution [30]. However, the government in some countries often avoided financial responsibility or squeezed public spending in LTC services, such as prevention and treatment of Alzheimer disease in the US [31] and home-based healthcare in the UK [32]. More worryingly, following the increasing number of disabled older people and the expanding financial burden of health care, there have been substantial variation in funding availability and payment rates for LTC between low- and high-income regions [33,14]. For instance, there was a remarkable disparity in LTCI benefits between civic villages in Japan. When public resource is typically limited, government might focus attention to certain types of LTC services. For example, the UK government spent 21% to 58% of total social care funding on home care services [34].

Thirdly, inconsistencies generate through organising the LTCI system and providing medical care & senior services. Although person-centered care has been widely adopted by the LTCI system in most developed countries, many LTCI policies ignored the interdependent relation between public health and social care, resulting in the serious shortage of community rehabilitation and nursing care services [35]. Furthermore, re-assessment of people’s disability and fragmentation of public healthcare services often caused extra administrative cost [36]. Apparently, there needs to be a expanded, specialized and diversified supply system for the delivery of different LTC services. This, however, have not been generally accepted or respected by all interest groups. As a consequence, the fragmented supply system led to inefficiencies and poor effectiveness in LTC [37,38].

Fourthly, there is no unified standard on public-private partnership (PPP) and administrative capacity for the provision of LTC services. The LTCI systems across countries suffer from some same defects [39,40]: firstly, services for older people are provided by different departments or institutions; secondly, policies on healthcare services often conflict; thirdly, different approaches are taken to provide immediate treatment and LTC. In essence, the LTC system consists of a range of services and assistance, which require partnership working between different organizations including public- and private-sectors as well as effective coordination between skilled professionals, in order to meet the varying needs of all disabled people [41,42]. These essential requirements pose significant challenges to the management and operation of the LTC system. Specifically, how local government promotes PPP plays a significant role in achieving successful LTC services [43]. For example, the UK government developed a good relationship with private sectors under the slogan of big society small government for the delivery of LTC, with 81% of home-based care being provided by commercial sectors in 2011 rising from 5% in 1993 [44]. By contrast, both Holland [45] and Germany [46] did not establish a national market system for the supply of LTC services, leaving predominant responsibilities on public sectors.

Regional and local differences have exerted considerable impact on access to LTC services and benefits [47]. To address this, many countries have made some adjustments to the existing LTCI system. However, there is still the fragmentation of responsibilities and policies for LTC provision. Policy fragmentation means a policy system with logical disjunction between policy values, policy objectives, policy practices, which leads to negative effects on functional effectiveness of public policy and organizational coordination in policy implementation [18]. It is mainly caused by fragmented governance, in which a large number of subnational administrative units are created and they have their own administrative capacity and interest groups [19,20]. In brief, the extent of government fragmentation has critical implication for policy fragmentation [21].

Given the geographical and institutional fragmentation of LTC provision, the WHO introduced the integrated care for older people (ICOPE) approach to build a person-centred LTC system and improve intrinsic capacity in older people for healthy ageing [48]. Integrated care is defined as services, such as prevention, treatment and rehabilitation, being managed and delivered across various levels and sites within and beyond the health sector to meet diverse needs of people throughout their life span [49]. In this regard, achieving integrated care requires the involvement of multiple levels and sites, which can be divided into system (macro), service/organisational (meso) level and clinical (micro) level [50]. In the context of LTC provision, the ICOPE approach supports the integration of health services and social care by promoting inter-organisational collaboration in different forms at and beyond the macro, meso and micro level (e.g. relationships between partner organisations or between different professionals) [51]. It provides the potential for innovation of LTC delivery and sustainability of the healthcare system.

A number of countries have managed to implement ICOPE in care settings and to improve coordination between health and social entities [47]. Its effectiveness, however, remains inconsistent. In practice, LTC continues to be funded by multiple financing sources and provided by partner organisations at different levels from the macro to the micro [52]. Since the provision of LTC can be regulated at national, regional and local levels, there is a vertical split of responsibilities between different governance levels [53]. Also, responsibilities for LTC delivery are often shared horizontally by public organisations and private providers [47]. Therefore, implementation of integrated LTC for older people can be achieved in two operational dimensions, directionally and functionally [54]. The directional integration can be promoted vertically as well as horizontally, with the former coordinating partners along the chain of LTC provision (e.g. integrating primary with secondary care) and the latter coordinating organisations at the same level (e.g. integrating public health with social care) [55]. The functional integration gives attention to coordination of responsibilities between organisations, between professions, and between medicals. Valentijn et al. [56] further suggested to develop functional coordination across policymakers (system integration), administrators (organisational integration), professionals (professional integration), and practitioners (clinical integration). Additionally, from the perspective of administration, whether decisions can be made by authorities independently places heavy impact on the effectiveness of LTCI policy integration [57].

**Figure 1:**
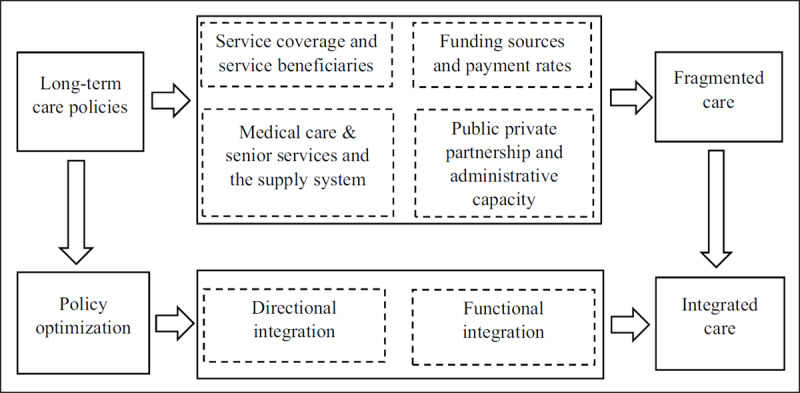
Theoretical framework for policy integration on LTCI.

Based on fundamental principles and intrinsic elements of the ICOPE approach, a theoretical framework is evolved in this study to shift from fragmentation to integration of LTC systems. It will explore the fragmented features of current LTCI policies and initiatives and to develop a strategy for integration and optimization of LTC provision (***Figure 1***).

## Materials and methods

### Sampling

**Figure 2:**
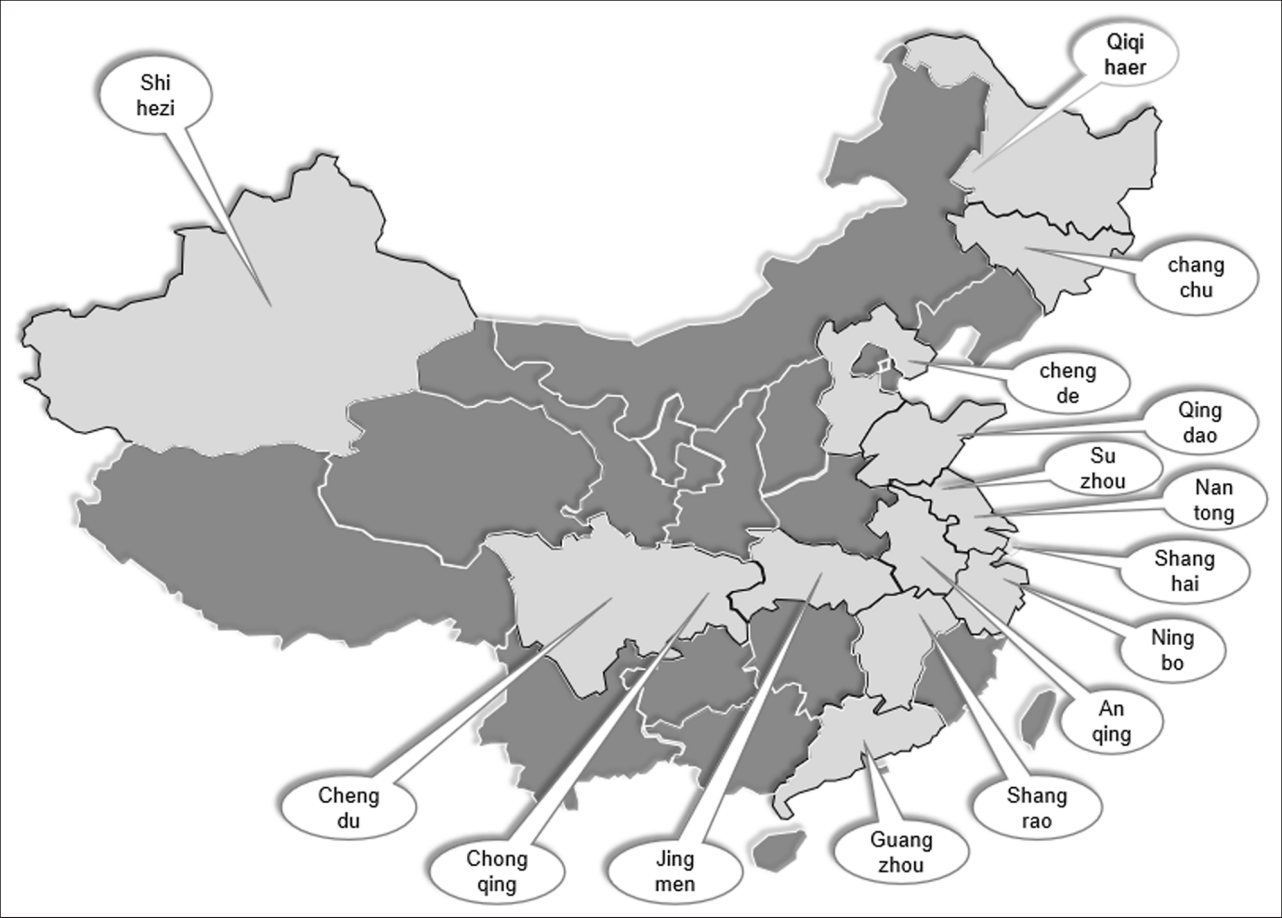
The geographical position of pilot cities in China.

This study adopts a systematic review that supports evidence-based practice and is often applied in the field of healthcare [58]. Differentiated from the traditional literature review, the systematic review aims to identify all relevant evidence and provide comprehensive synthesis of the knowledge [59]. The 2016 guidance initially designates 15 cities to pilot the LTCI programme across China, including Chengde, Changchun, Qiqihar, Shanghai, Nantong, Suzhou, Ningbo, Qingdao, Guangzhou, Anqing, Shangrao, Jingmen, Chongqing; Chengdu, Shihezi. Given the nature of the research aim, only these 15 cities can serve as primary data sources. Therefore, purposive sampling was used for systematic analysation as it provides an opportunity to focus on a particular group and identify their themes and concepts in greater depth [60]. ***Figure 2*** shows the geographical positions of all pilot cities, among these, 7 are in Eastern China, 2 in Northeast China, 2 in Southwest China, 1 in North China, 1 in Central China, 1 in South China, 1 in Northwest China. In practice, cities of Qingdao, Shanghai, Changchun and Nantong have launched initiatives to explore LTCI between July 2012 and October 2015. On the whole, all these local authorities are experiencing an accelerate growth of older population with physical frailty and have provided a set of LTC services for the elderly.

### Data collection

The 2016 guidance sets the objective to establish the LTCI system for an aging population as well as to achieve social development and social sustainability. It sets up the following basic requirements: first, LTCI provides financial assistance to people who is no longer able to carry out basic tasks of daily life; second, LTCI mainly covers workers who have participated in employee basic medical insurance; third, pilot authorities are encouraged to establish different sources of funding for LTC; four, LTCI participants are considered to contribute to around 30% of the total cost of the LTC services. In practice, each pilot authority designed their own strategic policies to implement the LTCI programme in accordance with local economic development and the government’s capacity. The characteristics and performance of these policies are of great importance in expanding the LTCI system to the entire country. Therefore, all specific policy documents published on pilot authorities’ websites for the provision of LTCI was systematically collected and analyzed. ***Table 1*** showed features and priorities of local LTCI policies across the pilot cities.

**Table 1:** Policies on the implementation of LTCI across 15 pilot authorities.


CITIES	POLICY DOCUMENTS	GIVE POWER TO	PROVIDE SERVICES

Chengde	Opinions of establishing and implementing the long-term care insurance system for urban employees (Trial) Measures of Chengde Municipality to the management of long-term care insurance for home-based care of urban employees (Trial)	Chengde Human Resources and Social Security Bureau	Institutional care, hospital care

Changchun	Opinions of establishing the medical care insurance system for disable people Measures of Changchun Municipality to the implementation of medical care of disabled people (Trial)	Jilin Human Resources and Social Security Bureau	Unspecified

Qiqihaer	Measures of Qiqihaer Municipality to the implementation of the long-term care insurance (Trial) Regulations on implementing the long-term care insurance in Qiqihaer (Trial)	Heilongjiang Insurance Regulatory Bureau	Institutional care, nursing care, home care

Shanghai	Measures of Shanghai Municipality to the pilot of the long-term care insurance Regulations on implementing the pilot long-term care insurance in Shanghai Measures of Shanghai Municipality to unified need assessment and service management for elderly care (Trial) Measures of Shanghai Municipality to the settlement of long-term care insurance (Trial)	Shanghai Municipal Human Resources and Social Security Bureau Shanghai Civil Affairs Bureau Shanghai Finance Bureau	Community-based home care, institutional care, hospital care

Nantong	Opinions on establishing the basic care insurance system (Trial) Regulations on implementing the basic care insurance Opinions of establishing the unified basic care insurance system across the municipality	Nantong Human Resources and Social Security Bureau	Home care, institutional care, hospital care

Suzhou	Opinions of implementing the pilot long-term care insurance Measures of Suzhou Municipality to the administration of social basic medical insurance Daily care services and their eligible criteria for the long-term care insurance in Suzhou	Suzhou Human Resources and Social Security Bureau	Hospital care, institutional care, community-based home care

Ningbo	Scheme of Ningbo Municipality on piloting the long-term care insurance Regulations on implementing the pilot long-term care insurance in Ningbo Measures of Ningbo Municipality to the pilot of disability assessment for the long-term care insurance	Ningbo Human Resources and Social Security Bureau Ningbo Finance Bureau	Institutional care, nursing home care

Anqing	Opinions of implementing the pilot long-term care insurance for urban employees in Anqing Measures of Qnqing Municipality to the implementation of long-term care insurance for urban employees	Anqing Human Resources and Social Security Bureau Anqing Finance Bureau	Institutional care, home care, disabled dependent care

Shangrao	Scheme of piloting the long-term care insurance system across the city	Shangrao Human Resources and Social Security Bureau	Home care, self-care, door-to-door care

Qingdao	Regulations on implementing the long-term care insurance in Qingdao (Trial) Interim measures of Qingdao Municipality on the long-term care insurance	Qingdao Human Resources and Social Security Bureau Qingdao Department and Reform Commission Qingdao Civil Affairs Bureau Qingdao Finance Bureau Qingdao Insurance Regulatory Bureau	For persons with disabilities: medical care, hospital care, door-to-door care, patrol care For persons with dementia: LTC, daily care and short-term care in institutional settings

Jingmen	Regulation on implementing long-term care insurance in Jingmen (Trial) Measures of Jingmen Municipality to the implementation of the long-term care insurance (Trial) Measures of Jingmen Municipality to the administration over the designated hospitals of long-term care insurance in (Trial)	Jingmen Human Resources and Social Security Bureau	Home care (full-time and part-time), institutional care, hospital care

Guangzhou	Opinions of Guangzhou Municipal Government about the implementation of the pilot long-term insurance system Measures of Guangzhou Municipality to the implementation of the pilot long-term insurance	Guangzhou Human Resources and Social Security Bureau Guangzhou Department and Reform Commission Guangzhou Civil Affairs Bureau Guangzhou Finance Bureau Guangzhou Insurance Regulatory Bureau	Integrated nursing care, door-to-door care

Chongqing	Opinions of piloting the long-term care insurance system in Chongqing	Chongqing Human Resources and Social Security Bureau	Institutional care

Chengdu	Scheme of piloting the long-term care insurance system in Chengdu Regulations on implementing long-term care insurance in Chengdu (Trial) Opinions of expanding the pilot scope of implementing the long-term care insurance system	Chengdu Human Resources and Social Security Bureau	Institutional care, home care

Shihezi	Opinions of establishing the long-term care insurance system (Trial) Regulations on implementing long-term care insurance in Bashi shihezi (Trial)	Shihezi Human Resources and Social Security Bureau	Institutional care, home care


### Data analysis

An exploratory data analysis approach was employed to look at local LTCI systems, including service coverage, service beneficiaries, funding sources, eligible criteria, types of medical nursing care, the supply systems, PPP, and administrative capacity. NVivo was adopted to analyse and compare policy documents across the pilot authorities, thematic analysis was used to identify and report patterns within these qualitative data.

## Results

Following over three years of implementing the LTCI programmes, some pilot authorities such as Qingdao and Chengdu have made significant progress. However, fragmentation of LTC for the elderly still remains. ***Table 2*** presents the features and characteristics of LTCI policies across the 15 pilot authorities. The coverage of LTCI services is found in many variations, ranging from small, medium to large. 53.3% of local authorities provided basic medical insurance just for urban employees (small), compared with 40% for urban employees and urban-rural residents (large) and 6.7% for urban employees and residents (medium). About who are eligible for LTC services, in 13 out of 15 local authorities, only a small group of people with several disabilities were qualified for LTCI. The remaining 2 authorities expanded the qualification to a large size of populations with several and moderate disabilities. Unfortunately, there is no single authority introducing LTC services for all older people, including those with mild disability. The multiple budget sources for LTC services started to prevail. Except for 20% of local authorities using the social medical insurance fund solely, the remaining authorities set up two (40%) or three and more (40%) grant schemes, including medical insurance funds, financial assistance, employers’ contributions, personal payments, and welfare lottery funds. The 2016 guidance defines the standard insurance contribution to LTC services as 70% of the total cost. However, only one third of local authorities set a higher payment rate than this standard; almost half (46.7%) of them has lowered their service cost.

**Table 2:** Characteristics and features of LTCI policies across 15 pilot authorities.


CITIES	COVERAGE			SERVICE BENEFICIARIES			FUND SOURCES			PAYMENT RATES			MEDICAL CARE & SENIOR SERVICES			SUPPLY OPTIONS			PUBLIC PRIVATE PARTNERSHIP		BENEFIT SCOPE		
							
	SMALL	MEDIUM	LARGE	SMALL	MEDIUM	LARGE	ONE	TWO	THREE/OVER	LOW	STANDARD	HIGH	ONE	TWO	THREE	ONE	TWO	THREE	SINGLE RESPONSIBILITY	JOINT WORK	SMALL	MEDIUM	LARGE

Chengde	√			√					√		√		√				√		√		√		

Changchun		√		√			√					√		√		√			√				√

Qiqihaer	√			√				√		√			√				√		√		√		

Shanghai			√	√					√			√			√		√		√		√		

Nantong			√	√					√	√			√				√			√			√

Suzhou			√	√				√		√					√		√			√			√

Ningbo	√			√			√			√			√				√			√	√		

Anqing	√			√				√		√			√					√		√			√

Shangrao	√			√					√	√			√					√		√			√

Qingdao			√		√			√				√		√				√		√		√	

Jingmen			√	√					√			√			√		√			√			√

Guangzhou	√				√		√					√			√		√			√			√

Chongqing	√			√				√		√			√				√			√	√		

Chengdu	√			√				√			√		√					√		√		√	

Shihezi			√	√					√		√			√				√	√		√		

**Tatol** **(%)**	**8** **(53.3%)**	**1** **(6.7%)**	**6** **(40.0%)**	**13** **(86.7%)**	**2** **(13.3%)**	**0** **(0.0%)**	**3** **(20.0%)**	**6** **(40.0%)**	**6** **(40.0%)**	**7** **(46.7%)**	**3** **(20.0%)**	**5** **(33.3%)**	**8** **(53.3%)**	**3** **(20.0%)**	**4** **(26.7%)**	**1** **(6.7%)**	**9** **(60.0%)**	**5** **(33.3%)**	**5** **(33.3%)**	**10** **(66.7%)**	**6** **(40.0%)**	**2** **(13.3%)**	**7** **(46.7%)**


There are many inconsistencies and inequities in the types of benefits. Medical care & senior services normally consist of daily care services and daily care related nursing & rehabilitation services. The results found that 73.3% of local authorities simply provided the first type (53.3%) or the second type (20.0%) for older people in need of LTC. Only 4 local authorities included both types of services to be covered by benefits. National government encourages local authorities to integrate institution-, community-, home-based facilities for the supply of LTC services. However, this proposal has not been widely accepted, just one third of local authorities have done it. The rest authorities relied on either the institution (6.7%) or a combination of institutions and communities (60%). The introduction of private sectors enables older people to select service providers and to improve service effectiveness. 66.7% of local authorities have developed joint work between commercial insurance companies and social medical insurance institutions, with the former being responsible for the insured persons’ requirements and the latter carrying out supervision over the procedures. On the other hand, there are still 5 authorities just empowering a public management institution for the delivery of LTCI. With respect to the benefit scope, there are two opposite ways at the local level. 40% of pilot authorities only insured LTC services, while another 46.7% authorities expanded LTCI to other health-related services including medicines, treatment, assistive equipment, care beds, nursing care and so on.

## Discussion

Research results showed that local policies for the implementation of LTCI in China were fragmented with a range of existing issues. Firstly, because of an aging population and their longevity extension, there was a significant shortage of LTC services for older people, leading to a new social risk [61]. Secondly, local government was empowered to carry out their own policies in accordance with local aging process and economic development [62]. Thirdly, China’s social policy implementation is often confined to path dependence. Reforms of social security system, including basic living allowance, low-rent house, endowment insurance and medical insurance, has been piloted by local government since 1990s [63,64]. After summarising and analysing the characteristics of successful local pilots, central government then published national policies to promote their implementation across the country. In order to protect older people’s rights and to ensure effective use of LTC resources, local authorities require integrating policies in a directional and functional way.

Based on the theoretic framework for policy integration, service coverage and service beneficiaries are the key benchmark against which performance of LTCI policies is assessed. On average, only 40% of residences have been covered by LTCI services, with only 13% of moderately disabled older people except for severely disabled older people receiving LTC services. There is a need to expand LTCI services, covering all residences in urban and rural areas [65]. Besides, older people with mental and physical disability, no matter moderately and severely, should receive health care and nursing services [66]. This requires central government to compare local LTCI programmes, identify positive experiences, and integrate local priorities into national policy for multiple functionalities [67].

Funding streams and payment rates setting have significant effects on LTC insurability and sustainability. Similar to other countries, the primary source of financing LTC services in China is national expenditures. However, evidence from the pilot authorities showed that direct public subsidies towards LTC were very limited. To expand LTCI programmes nationally, it is necessary to build multiple streams of funding from individuals, enterprises, public resources and private donations [13]. Local government should pool all these financial resources to achieve a better balance between the public and private funds [11]. For those vulnerable elderly households, the government should provide means-tested benefits along with LTCI to improve social governance [68]. Meanwhile, to avoid any moral hazard effects and maintain relatively appropriate social protection, a 10% to 30% co-payment can be introduced like German and Japan for individuals to take certain responsibility for LTC services. In this regard, local government should seek coherent cross-sectoral policy instruments to perform their cooperative functions.

Types of medical care & senior services and their supply systems are important features of an effective LTCI policy framework. ***Table 2*** found that only one third of pilot authorities have provided home-based LTC. However, most older people desire to live longer in their own homes; aging in place contributes to not only a better support from family but also a reduced burden to the social care system. In fact, favourable effects of LTC policies in many countries are largely dependent upon informal family caregiving [69]. Therefore, it is vital to develop home-based LTC services, especially daily care services and rehabilitation therapy services [70,71]. This requires local authorities to create interdependencies between different policies on ageing at home, aging in community and aging in institution, and then coordinate them. In this coordination process, there should be combination of formal services and informal care with caring as a focus, nursing as a priority and medicing as a supplement [72]. More specifically, community medical resources are delivered to support older people stay healthy at home; sickbed is installed in the house for family members with severely chronic diseases; severely disabled older people are allowed to live in care-based or nursing-based institutions [73].

PPP and administrative capacity are fundamental to strength the LTCI system. In western countries, the provision of LTC services has traditionally been a cooperation between profit-making sectors and non-profit sectors. For instance, in England non-profit institutions and private for-profit have been cooperated in LTC provision for years, with 89% of care at home and 94% of beds in residential settings being provided by private sectors [47]. Similarly, in Ireland, the severe shortage of public resources brought about marketisation and privatisation of LTC services, with around 75% of LTC services contributed from private commercial providers [74]. In contrast, findings from pilot authorities showed that PPP for LTC provision in China was very limited and only commercial insurance companies were involved on the private side. To address this, it is important for national and local government to integrate LTC policies, to coordinate different functional departments, and to encourage private and non-governmental organizations working in partnership with public institutions.

## Conclusion

The introduction of LTCI made innovative changes to the provision of aging services in China. This social insurance mode is selected because of the existing five social insurance systems in China, just like the LTCI act implemented in Germany and Japan [75]. The pilot of LTCI policies is a crucial approach to social governance in China.

The LTCI programmes have been piloted for five years across China and produced some substantial progress in the support for the elderly. However, there are still some serious problems. This study established a clear picture of policy fragmentations in key aspects, including service coverage, service beneficiaries, funding sources, payment rates, medical services & senior services, supply options, PPP and management capacity. The major issue with the LTCI system is not only the cost but budget allocation [19]. To address this issue, there needs to be a range of strategic initiatives.

First, it is necessary to integrate service concept, which requires the vertical integration of policy makers and the horizontal integration of service providers. In other words, attentions should transfer from the life course to a consistent preventive action [76], from forced care to independent living [77], from traditional daily care to a combination of daily care and rehabilitation services [40].

Second, it is important to pool all financial sources together. This requires the integration of funding from both the civil administration and the disabled persons’ federation, the optimised allocation of various welfare subsidies for the elderly and the disabled, and the avoidance of full reliance on medical insurance funds [78].

Third, another key thing is to integrate the process of service delivery. The LTC system should strengthen the horizontal integration of rehabilitation services and hospice care, establish a competitive PPP service system [66], especially induce private suppliers and arrange a monitoring and managing system like Israel [79].

At last, there needs to be an integration of service beneficiaries. The sustainability of the LTCI system is subject to “who will benefit”, but not all disabled elderly people can benefit in China. Therefore, there is usually a compromise. More importantly, formal care and informal care should be integrated to establish a service user-oriented delivery system and to meet the LTC needs of older people with chronic diseases or severe disabilities [80]. This can secure equity and efficiency in LTC interventions.

## Figures and Tables

**Table:** 

WHO:	World Health Organization
LTC:	long-term care
LTCI:	long-term care insurance
ICOPE:	integrated care for older people
PPP:	public-private partnership
